# Supplementary material for: CRISPECTOR provides accurate estimation of genome editing translocation and off-target activity from comparative NGS data
Source: Nat Commun. 2021 May 24;12:3042. doi: 10.1038/s41467-021-22417-4 (PMC8144550; doi:10.1038/s41467-021-22417-4)
Supplement: Supplementary file 1 — Supplementary Information [file 41467_2021_22417_MOESM1_ESM.pdf]

Supplementary Information

Supplementary figures

SiteName	AmpliconReference	gRNA	OnTarget	ForwardPrimer	R_PRIMER	TxInput1Path	TxInput2Path	MockInput1Path	MockInput2Path	DonorReference
RAG2_1	GATCTCATTTTGGATGTA	AGGGAAGCCTGG	FALSE	GATCTCATTTTGGA	TGATTTCCCTGAGACTGCTAA					
RAG2_10	CTCAAGTTCAGTGCTGTA	AGAGAAGTCTGA	FALSE	CTCAAGTTCAGTGC	TAGGAAAGTGTGGATCTTGC					
RAG2_11	TCTTAGAGAGAATGGGC	AGAGAAGCAAG	FALSE	TCTTAGAGAGAATG	CGCTCATTTCTTGACATAGTTTC					
RAG2_12	GGCAATTCTAAAATTTCA	GTAGAAGGCTGA	FALSE	GGCAATTCTAAAAT	GCATTGTTCAGTGTCAAAAATCA					
RAG2_13	TCCTCTCTACTACTCTGCT	ATAGAAGTCTTG	FALSE	TCCTCTCTACTACTC	ACTTCTGCTTTCTGCCTAGTA					
RAG2_14	GTTCAGTCCAATGAGCCA	TGTGAAGACTGG	FALSE	GTTCAGTCCAATGA	CCTGTCAGTGTACCAACAAAA					
RAG2_15	GTTCTGACAAACTGACAA	GAGGAGACTTGG	FALSE	GTTCTGACAAACTG	GGAAACTGAAGCAGACCATTT					
RAG2_16	ATGATTCTTGCTACAGCC	CCAGAAACCTAG	FALSE	ATGATTCTTGCTAC	GGGATCAGTGTTGATTGGTT					
RAG2_17	TATGTGCCCTAATAAAGC	TGGGAAGCCTGG	FALSE	TATGTGCCCTAATA	GAATCAGCATTCTGTCCATTT					
RAG2_18	CAGGTTCTCCTTTGACCT	TGAGAAGGCTGA	FALSE	CAGGTTCTCCTTTG	CGCTTTTCTTTCTACATTCATGGTA					
RAG2_19	TGAAAAGTTGTTGACCAA	AGAGAAGCCGGC	FALSE	TGAAAAGTTGTTGA	TTTTGAAGCATGGCCTAGATAAA					
RAG2_2	GCCTTTTTGTCCAAAGAA	TGAGAAGCCTGG	TRUE	GCCTTTTTGTCCAA	CAGAAACTATGTCTCTGCAGATG					
RAG2_20	TGCATCTAGGACCCAGG	GGAAAAGCATGG	FALSE	TGCATCTAGGACCC	GGGTTCTTTTGCTGGGA					
RAG2_21	CAATGACAAGGACTAGTT	ACAGAAGCCTGG	FALSE	CAATGACAAGGACT	GGTATTGAATTATGTCTCAGTCCC					
RAG2_22	CATATACTGCCATTGTGC	GTGAAGCCTGGG	FALSE	CATATACTGCCATT	CATATGAAAATGCAGGCTAGA					
RAG2_23	ATTTTAGGCTTCTTTTTCT	AGAGAATGCTGG	FALSE	ATTTTAGGCTTCTTT	GTCAATGTGTGAGATCATCAGTTTT					
RAG2_24	ACATGCTCTGGATGATTA	ATTAAGCCCTGG	FALSE	ACATGCTCTGGATG	TAGATCAGATCTTCAAACAGACA					
RAG2_25	CTTATCTTGCCACACTTC	AGAGCAGCCTGG	FALSE	CTTATCTTGCCACA	GCAGATAAGCATTTCACAGGT					
RAG2_26	CCAATTAAGAATAAGTGA	TGAGAAACATGA	FALSE	CCAATTAAGAATAA	CTGTATCCATTCTTGAGTTATCGAA					
RAG2_27	TGGCTCAGTGATGTTCTC	TGGGAAGCCTGG	FALSE	TGGCTCAGTGATGT	CACAGGCAAAGAGACAGAAT					
RAG2_28	ATTTTCCCTCACTCACAC	AAGAGACCTGGG	FALSE	ATTTTCCCTCACTCA	AGATTGCAGGCTGAGAGATTA					
RAG2_29	ATCTACCAACCTCCAGAA	TGAGAATAATGG	FALSE	ATCTACCAACCTCC	GGTCAGGGTGTAGAAAGTTCTT					
RAG2_3	GTAACACTCCTTGTCACC	AGAGAAGCCAGA	FALSE	GTAACACTCCTTGT	GGAGAGTGGTGAGTTATAGGAAT					

Supplementary Figure 1 **Experiment configuration file**. SiteName, AmpliconReference, gRNA and OnTarget are mandatory. ForwardPrimer and ReversePrimer are inferred from AmpliconReference when not presented. TxInput1/2Path and MockInput1/2Path are full paths to multiple singleplex-PCR FASTQ files and should be left empty for an experiment with multiplex-PCR FASTQ files. DonorReference should be specified for on-target site, in the case of HDR/Donor experiment.

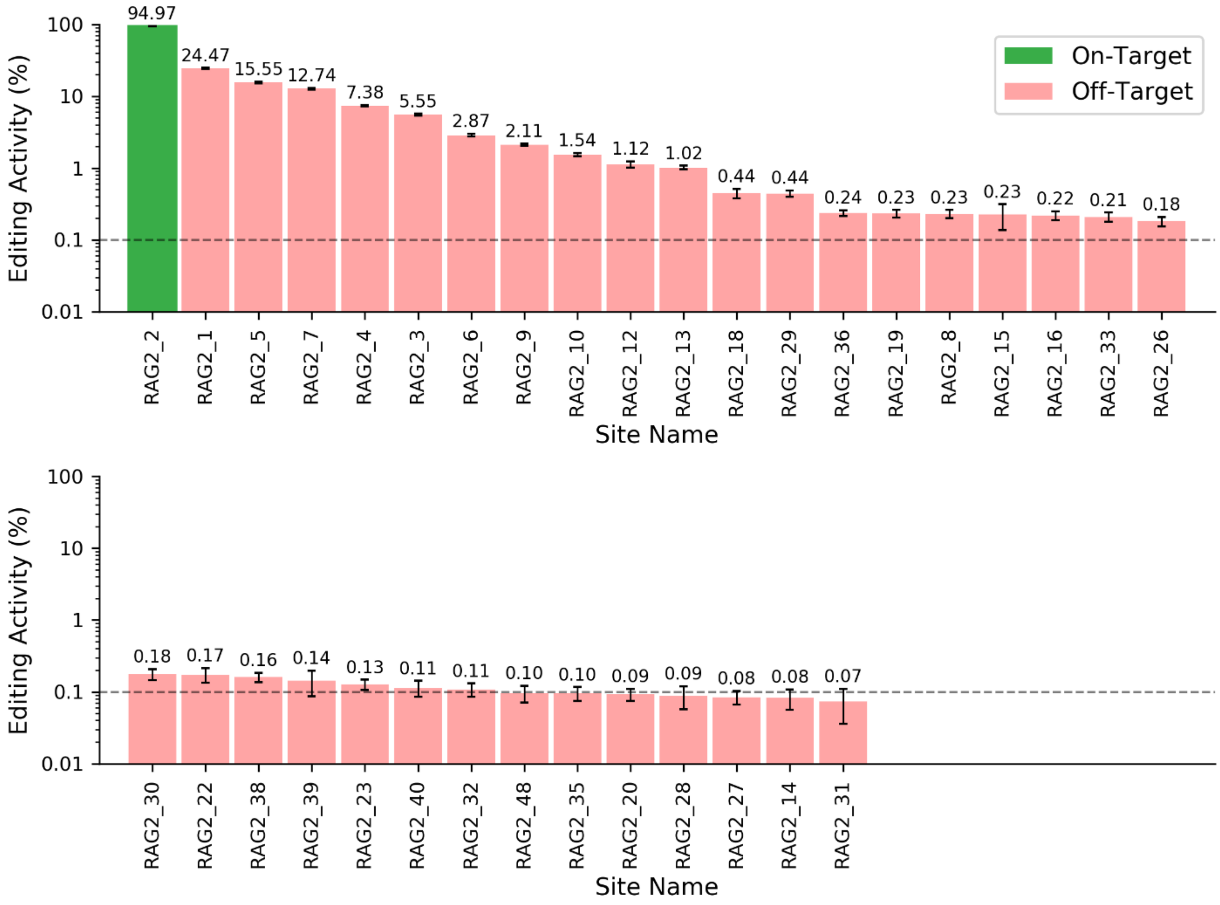

Supplementary Figure 2 ***RAG2* indel editing activity**. Editing activity for *RAG2*, XT 2p, in HEK293-Cas9 sample. Only off-target sites with a 95% confidence interval (represented as errors bars) extending above 0.1% editing rate are presented. The editing activity rate is presented above each bar. See Supplementary Figure 4 for *RAG1* indel results. Genomic locations of these sites are listed in Shapiro, J. et al, 2020[1].

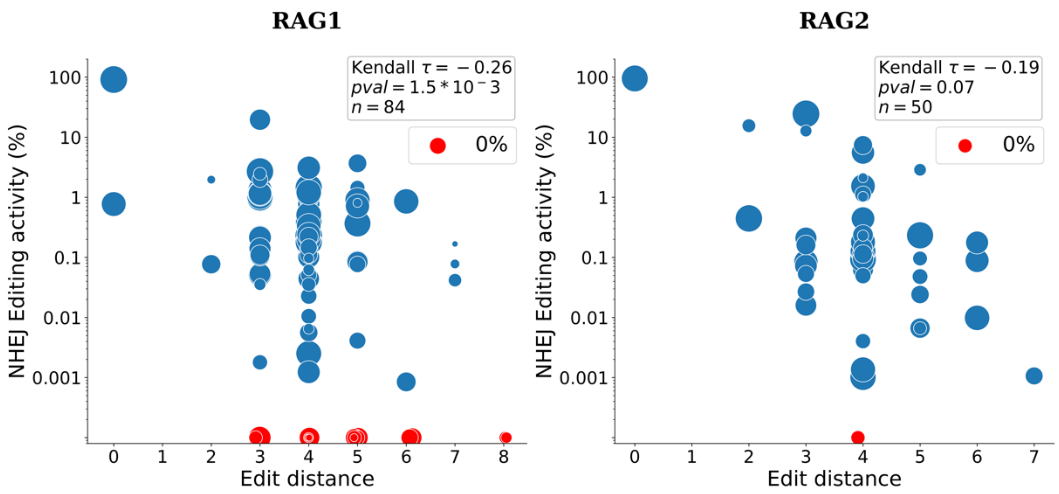

Supplementary Figure 3 **Off-target indel editing activity as a function of edit distance from the on-target sequence**. Data represents *RAG1* and *RAG2* on and off-target editing activity experiments in HEK293-Cas9 stable cells. Point sizes correspond to GUIDE-seq[2] results (larger point $\rightarrow$ higher GUIDE-seq read count). n represents the number of on- and off-target sites identified by the GUIDE-seq assay. Blue dots represent sites found to have editing activity, by the rhAmpSeq experiment. Red dots - sites with 0 observed editing percentage. Kendall τ and the corresponding one-sided p-values are calculated using standard methods[3].

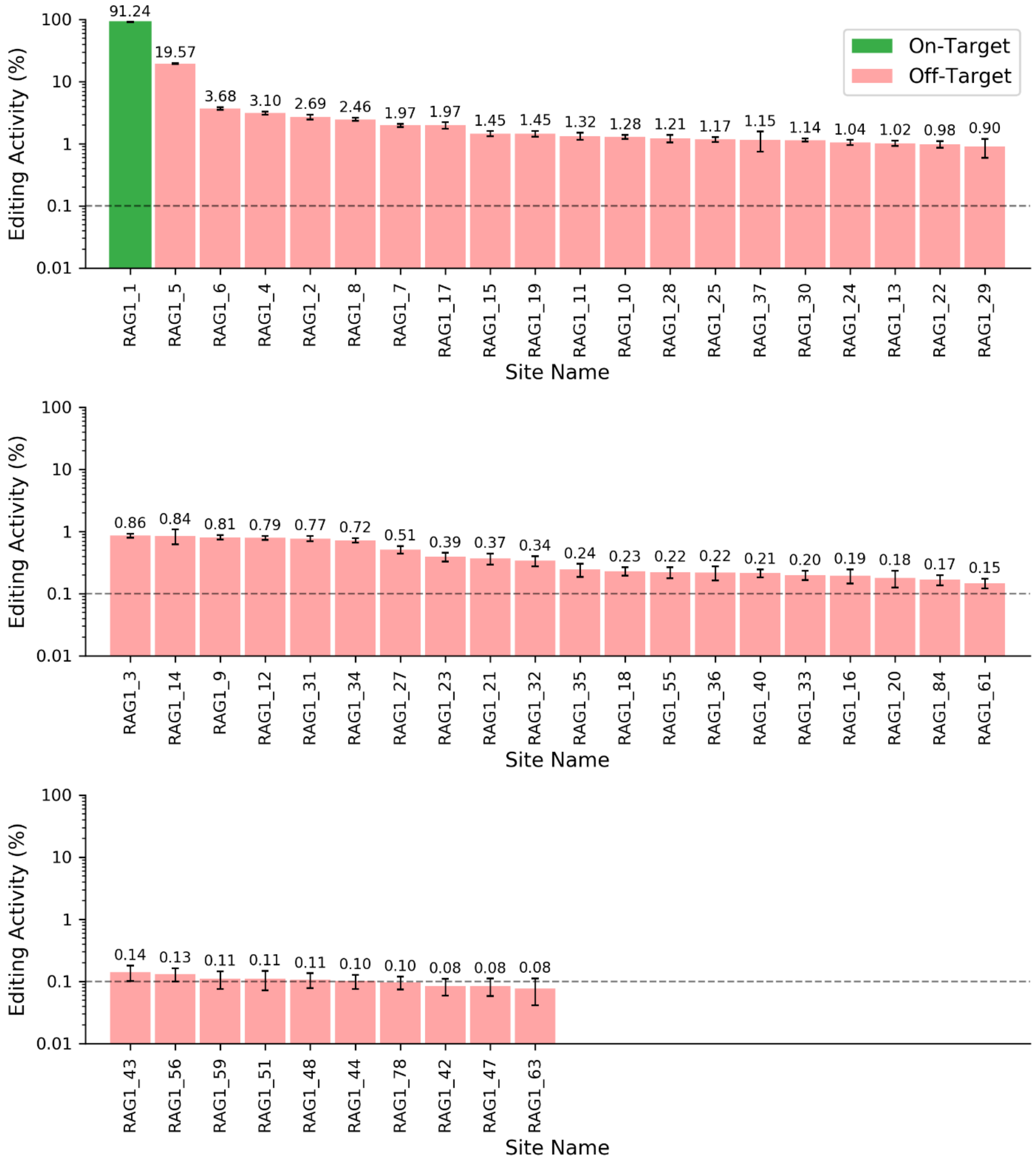

Supplementary Figure 4 ***RAG1* indel editing activity.** Editing activity for *RAG1*, XT 2p, in HEK293-Cas9 sample. Only off-target sites with a 95% confidence interval (represented as errors bars) extending above 0.1% editing rate are presented. The editing activity rate is presented above each bar. Genomic locations of these sites are listed in Shapiro, J. et al, 2020[1].

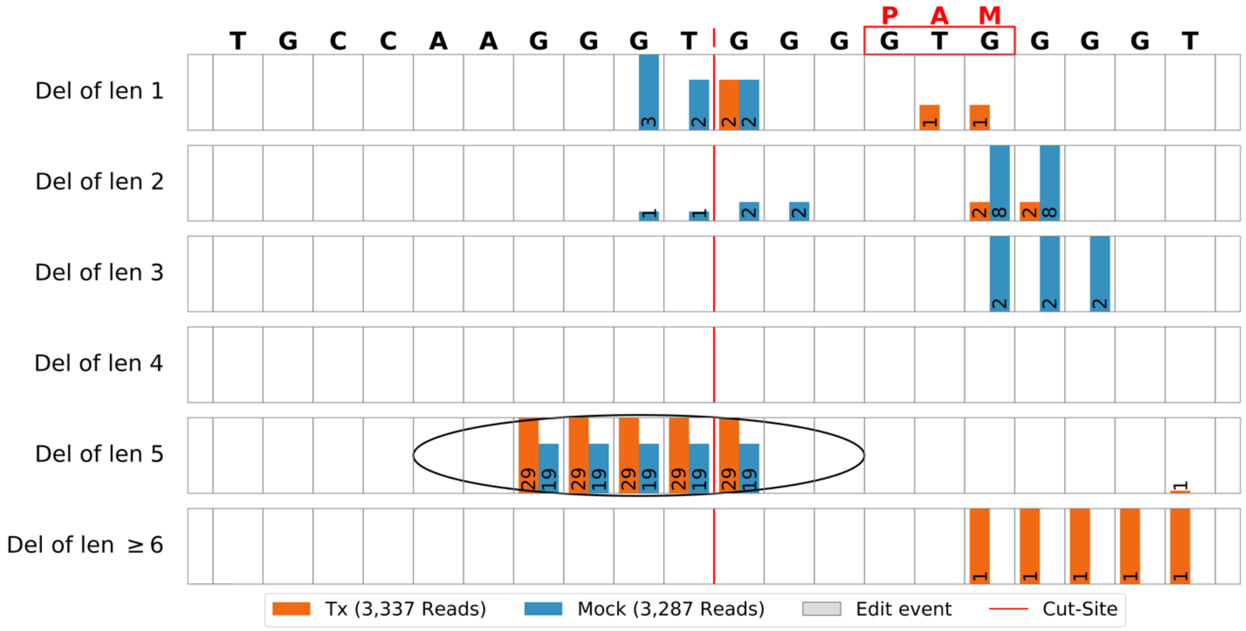

Supplementary Figure 5 **CRISPResso2 and ampliCan False Positive for Site 37 of *RAG1*, sg 1:2.5 no EE 4uM in CD34+**. The high rate of deletions of length 5 at the expected cut-site arises a CS2 error (Tx=29, Mock=19). Note the rate of deletions of length 2 outside the cut-site (Tx=2, Mock=8), with an opposite direction. Editing activity estimation – CRISPECTOR = 0%, CRISPResso2 = 0.136% and ampliCan = 0.317%. Note that the original genome coordinates correspond to a PAM that is different from NGG.

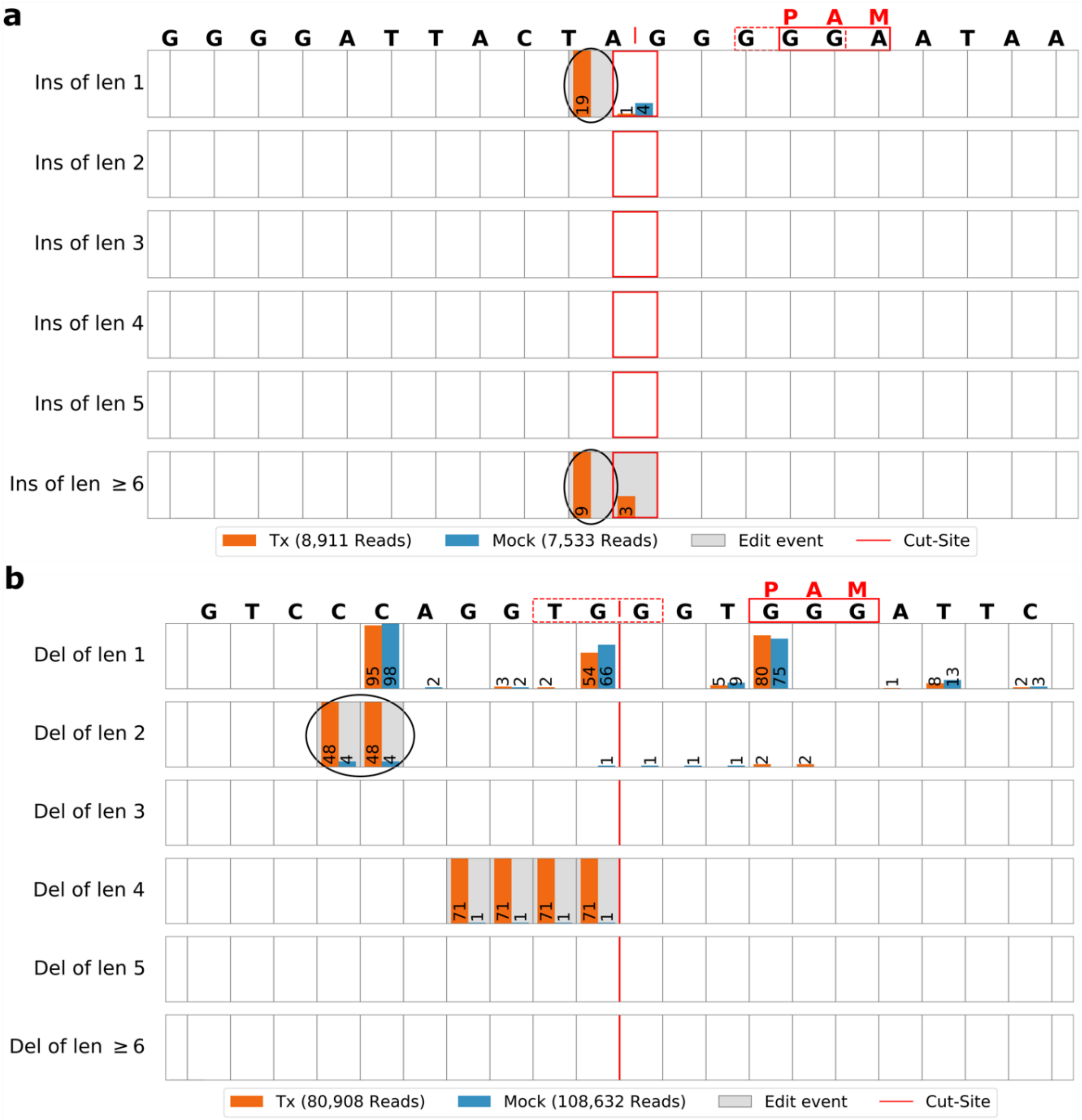

Supplementary Figure 6 **CRISPECTOR detects alternative cut-sites** (a) CRISPResso2 and ampliCan False Negative for Site iGS5 of *HPRT*, HiFi Cas9 in HEK293. We can find the alternative PAM (marked with dashed red) one base left to the original. CRISPECTOR observed 19 reads with insertion of length 1 and nine reads with insertion of lengths 34-68 at the alternative cut-site. Note that the original genome coordinates correspond to a PAM that is different from NGG. Probably, CRISPResso2 missed the insertions due to a narrow qualification window size. While ampliCan, due to high noise rate for deletions (not presented in this figure). Editing activity estimation – CRISPECTOR = 0.359% (*CI* ± 0.125%), CRISPResso2 = 0.065% and ampliCan = 0.095%. (b) CRISPResso2 and ampliCan False Negative for Site 61 of *RAG1*, XT 2p in HEK293-Cas9. We can find the alternative PAM (marked with dashed red) five bases left to the original. CRISPECTOR observed 48 reads with deletion of length 2 at the alternative cut-site. Probably, CRISPResso2 and ampliCan missed the deletions due to a narrow qualification window size. Editing activity estimation – CRISPECTOR = 0.147% (*CI* ± 0.027%), CRISPResso2 = 0.087% and ampliCan = 0.026%.

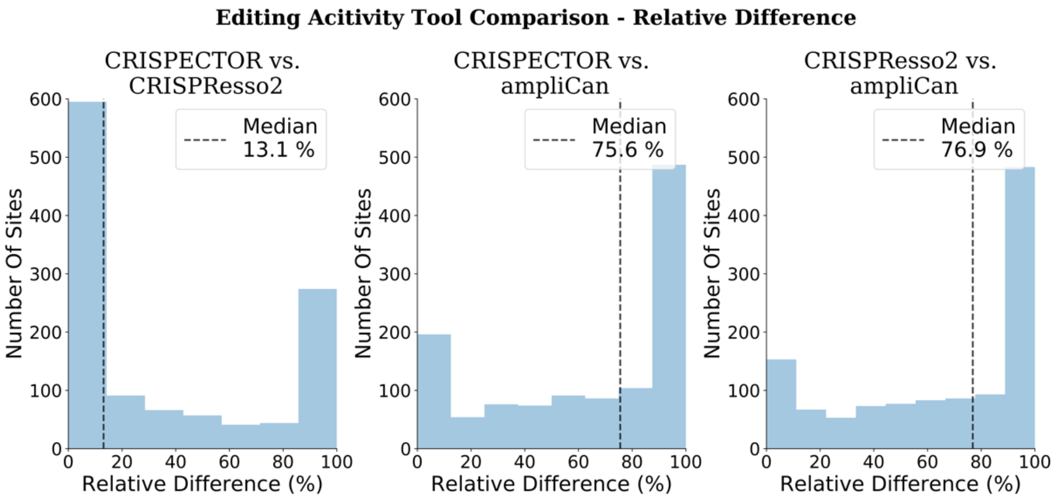

Supplementary Figure 7 **Editing activity Relative difference between the different tools**. Relative difference is defined as $\frac{|\hat{p}_1 - \hat{p}_2|}{max\ (\hat{p}_1, \hat{p}_2)}$ where $\widehat{p_1}$ and $\widehat{p_2}$ denote the estimated indel activity of the two examined algorithms. CRISPECTOR and CRISPResso2 produce similar results with a median of 13.1% relative difference. However, ampliCan estimation is notably more different from both CRISPECTOR and CRISPResso2. In particular, the ampliCan vs CRISPECTOR and ampliCan vs CRISPResso2 relative difference distributions have a high median of $\geq 75\%$. Moreover, in both these comparisons, we observe 39% of the sites to have more than 90% relative difference.

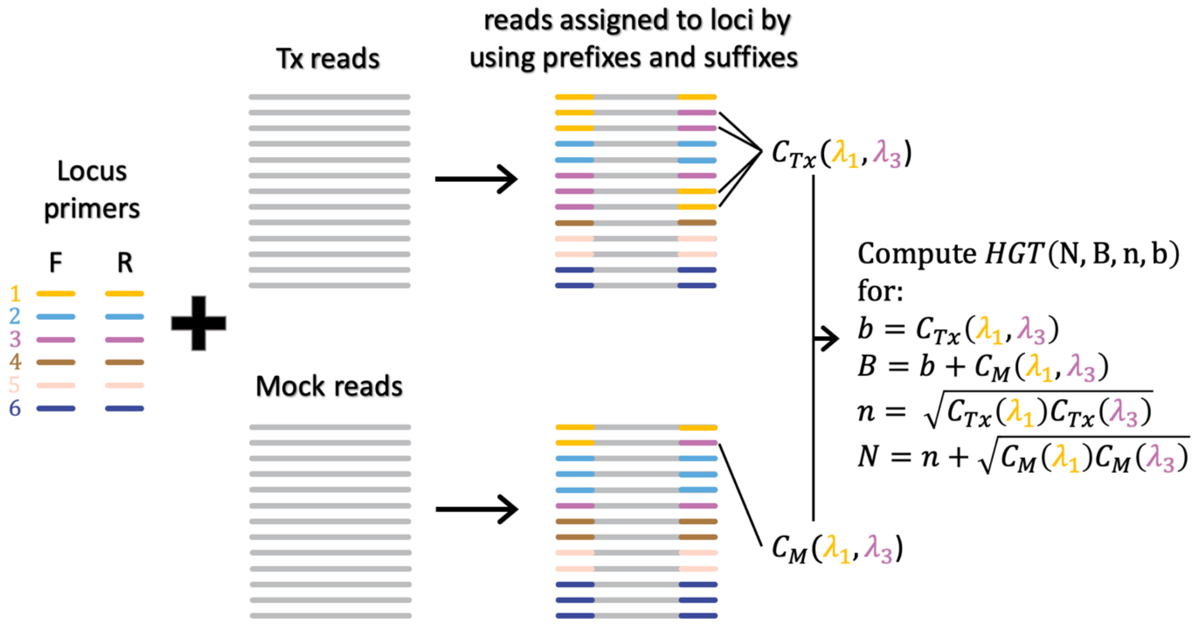

Supplementary Figure 8 **Translocation detection overview.** CRISPECTOR detects reads that are putatively originating from translocations by matching read prefixes and suffixes to forward and reverse primers. CRISPECTOR counts the number of putative translocation reads in Tx and M for each sites pair, $C_{Tx}(\lambda_1, \lambda_3)$ and $C_M(\lambda_1, \lambda_3)$ for sites λ_1 and λ_3 as demonstrated above. CRISPECTOR performs a hypergeometric test to determine whether translocation is likely to have occurred.

site A	site B	treatment reads	mock reads	treatment background	mock background	p_value	FDR
RAG2_1	RAG2_5	225	3	38652	42068	2.21126e-67	1.52577e-65
RAG2_1	RAG2_7	71	3	42370	50460	6.84218e-21	2.36055e-19
RAG2_1	RAG2_2	35	1	34004	49898	4.03561e-13	9.28191e-12
RAG2_1	RAG2_12	34	0	44710	48035	1.67111e-11	2.88266e-10
RAG2_1	RAG2_10	38	2	67789	75773	9.35965e-11	1.29163e-09
RAG2_1	RAG2_29	25	0	68762	76599	7.43892e-09	8.55475e-08
RAG2_2	RAG2_5	19	0	23729	35362	2.94869e-08	2.90656e-07
RAG2_2	RAG2_3	18	0	45719	66740	9.18292e-08	7.92027e-07
RAG2_3	RAG2_7	22	1	56967	67492	4.40471e-07	3.37694e-06
RAG2_2	RAG2_4	15	2	41453	64842	4.01708e-05	0.000277179
RAG2_19	RAG2_29	12	0	100415	115861	0.000100307	0.000629196
RAG2_3	RAG2_6	11	1	78090	97218	0.000972375	0.00559115
RAG2_19	RAG2_8	7	0	104527	138801	0.00269896	0.0138752
RAG2_2	RAG2_7	6	0	26012	42416	0.00301635	0.0138752
RAG2_10	RAG2_6	7	0	71084	92782	0.00289006	0.0138752
RAG2_16	RAG2_8	6	0	93054	135362	0.00457112	0.019713
RAG2_1	RAG2_6	10	2	58080	72685	0.0070345	0.0285518

Supplementary Figure 9 **An example of CRISPECTOR reads table for translocation detection**. The table shows the number of treatment and mock fusion reads between different target sites as well as the background numbers, the resulting p-value and FDR value, as computed from the hypergeometric null model. See Methods section for more details.

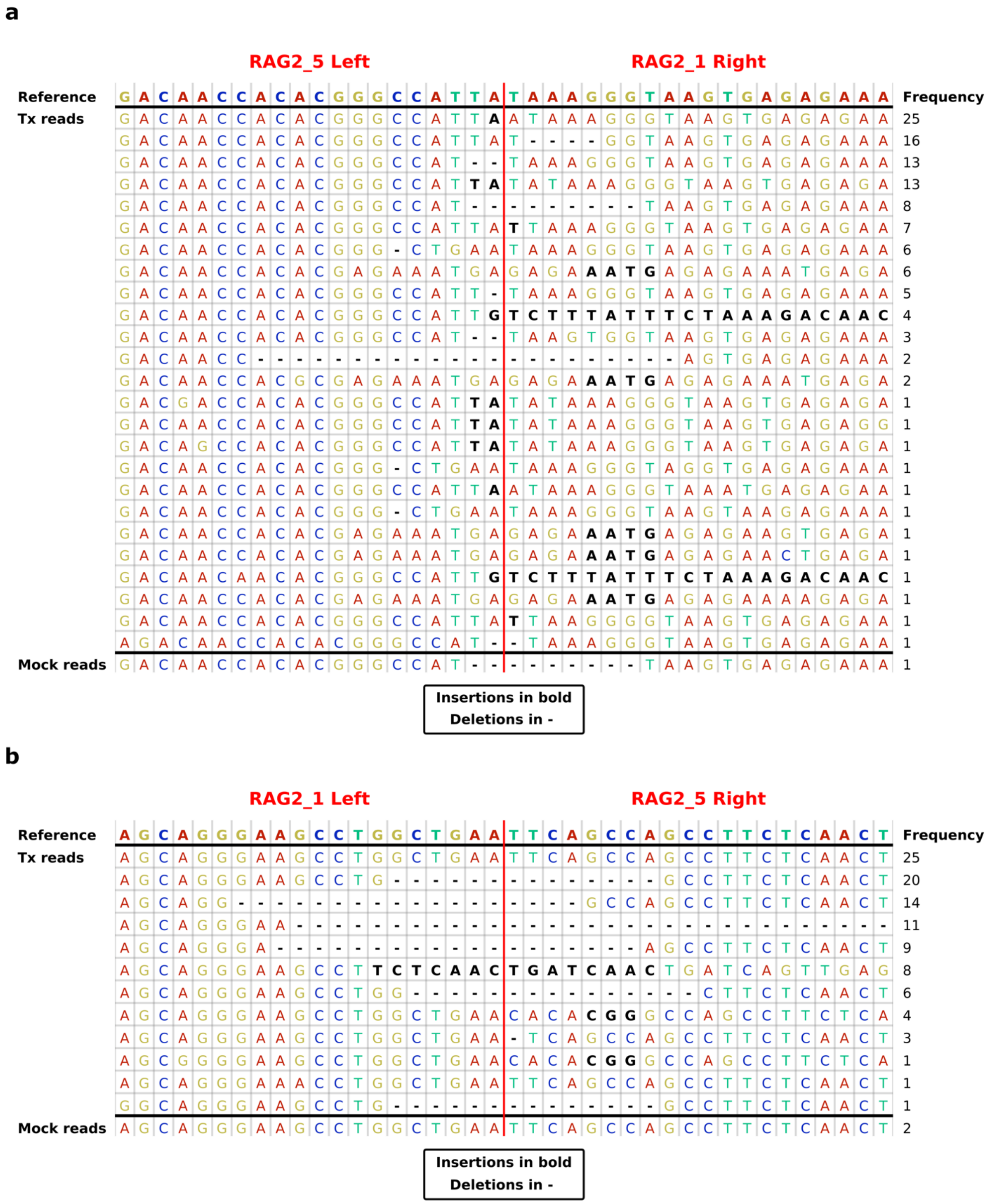

Supplementary Figure 10 **Translocation reads for off-target sites 1 and 5 for the *RAG2* experiment in HEK293-Cas9 stable cells.** Sequenced translocation read outcomes of the *RAG2* gene in HEK293-Cas9 cells. The red line depicts the cut site and it can be seen that the reads on either side of the cut site are *RAG2_1* and *RAG2_5*. Due to the fact that there are several different types of indel combinations, it is understood that these are actual translocation events and not PCR artifact. A and B represent the two types of detectable translocation possibilities in every pair. Bold letters represent insertions and dashes represent deletions.

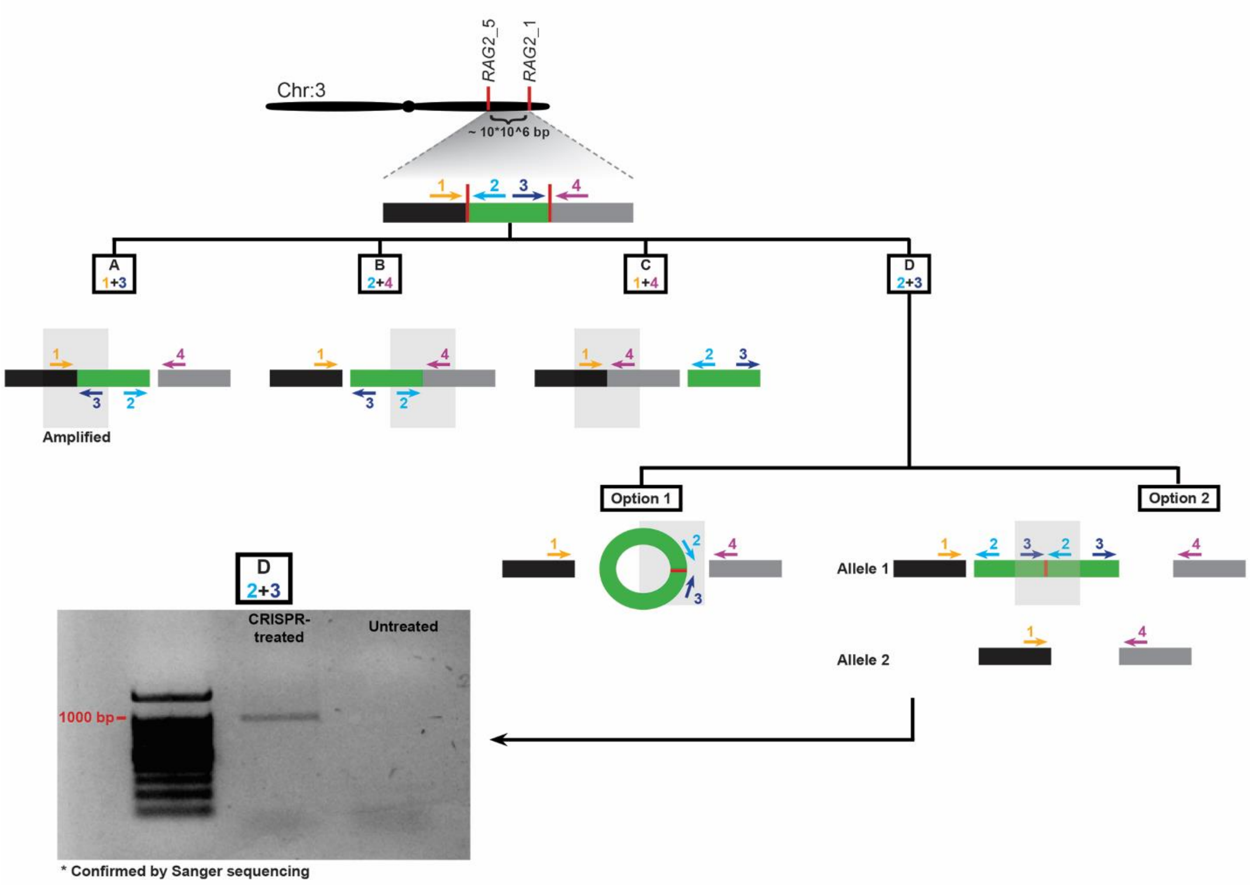

Supplementary Figure 11 **Schematic description of possible translocation/structural variation types and successful PCR amplification of these rare events.** Displayed is the most prevalent structural variation event amongst the *RAG2* (*RAG2_1+5*) which occurs between the two most efficiently edited off-target sites. To amplify the specific event there are 4 primer pair combinations that could be employed (A[1+3, causing an inversion], B[2+4, causing an inversion], C[1+4, causing a deletion], and D[2+3]). Primer combination D could give rise to two distinct resection events that both would be amplified simultaneously (option 1 [a centromere-free, circular, rearrangement] and option 2 [a rearrangement where both alleles are resected to one another creating a repeat sequence]). Each primer combination would amplify a different aberrant resection of these two off-target sites. Successful amplification of the D primer combination was achieved in the treated sample (CRISPR-treated lane) and not in the mock sample (untreated lane). This was further confirmed through Sanger sequencing. Source data is provided as a source data file.

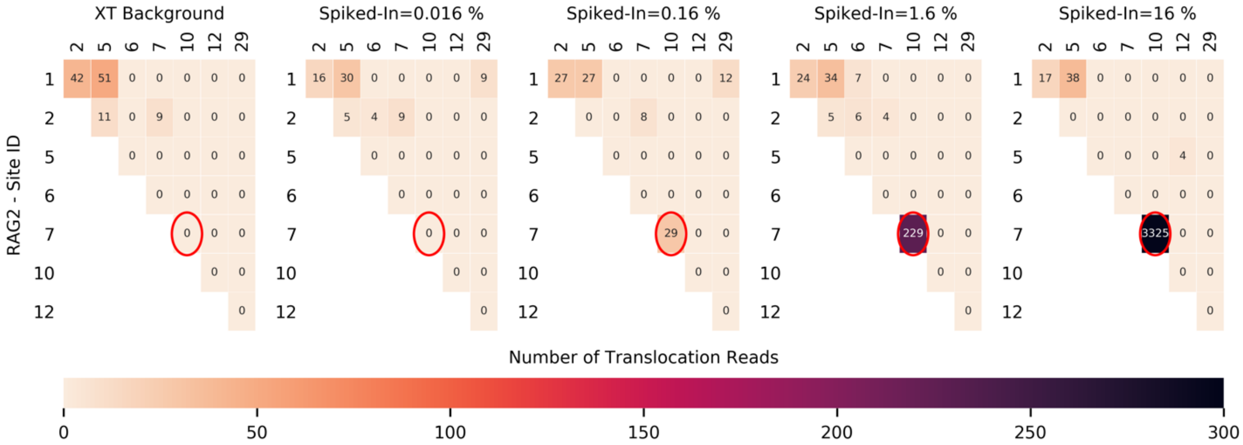

Supplementary Figure 12 **Validation of CRISPECTOR translocation detection process based on synthetic spike-in standards.** Edited *RAG2*, XT 2p, HEK293-Cas9 genomic DNA was spiked-in with serial dilutions (0%, 0.016%, 0.16%, 1.6% and 16%) of *RAG2_10/RAG2_7* synthetic translocation construct. Heatmaps of the detected translocation events at the various conditions are represented. *RAG2_10/RAG2_7* translocation reads are circled in Red. All translocation events with an (FDR corrected) p-value < 0.05 are presented in the heatmap with the associated read counts. p-values measured for 16%, 1.6% and 0.16% *RAG2_10/RAG2_7* spiked-ins are $< 10^{-300}$, $4.88 * 10^{-79}$, and $1.1 * 10^{-10}$, respectively**.**

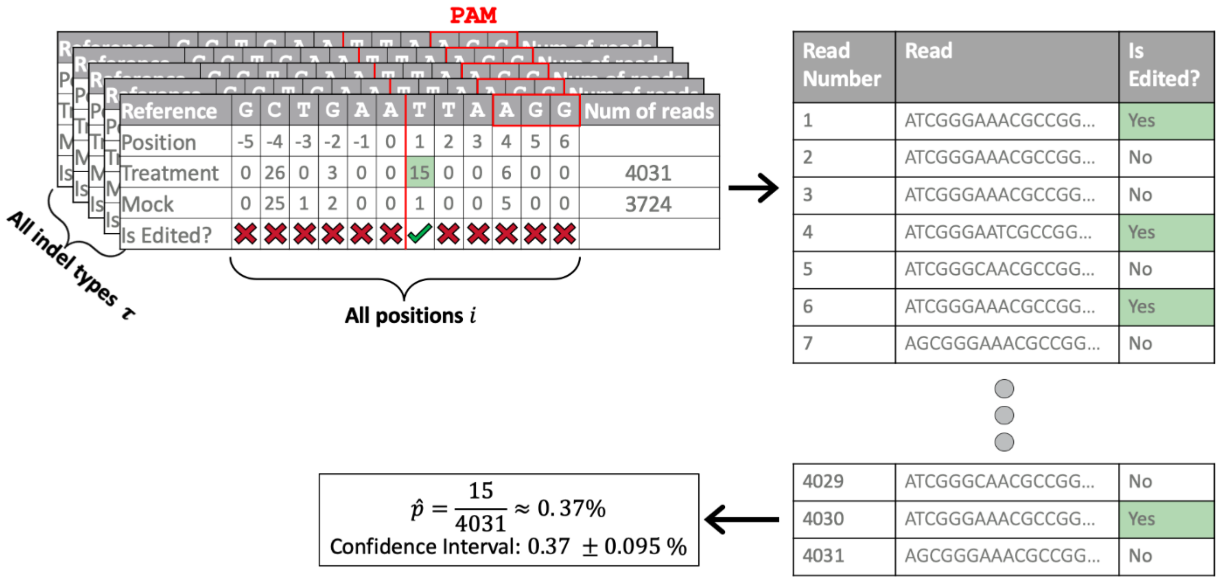

Supplementary Figure 13 **CRISPECTOR classification overview.** First, treatment and mock read lists are converted to indel type tables, where each table represent an indel type, τ, and each column represents a position on the reference locus, i. In the above example, 15 Tx reads and 1 mock read have the same indel type τ at the reference position 1 (relative to the expected cut-site). Then, CRISPECTOR applies a Bayesian inference classifier on every (τ, i) pair, to classify each pair as originating from an edit event or from background noise. Second, all Tx reads that were classified as positive in at least one pair (τ, i), are marked as edited reads. Finally, CRISPECTOR estimates the indel editing activity rate (e.g. 15 classified as edited out of 4031), as well as associate confidence interval to this estimation.

Supplementary Tables

	RAG1	*RAG2*	*AR*	*HPRT*	*EMX1*	Total
Number of off-target sites	83	49	49	35	10	226
Number of experimental configurations	6	6	3	3	10	-
Read depth per site and configuration: $\mu \pm \sigma$ in K	51 ± 40	40 ± 40	11 ± 9	12 ± 7	20 ± 33	-

Supplementary Table 1 **Experimental Data Summary**. Five different on-target genomic loci covering 226 off-target sites, under different experimental conditions. This yields a total of 1,161 instances.

	RAG1	*RAG2*	*EMX1*
single-centromeric	5	5	0
centromere-free	6	6	8
double-centromeric	6	6	0
Same chromosome	4	5	1

Supplementary Table 2 **The prevalence of the different fusion configurations in our experimental data.** All four possible configurations of translocations (Figure 3a) for different pairs of loci were observed in our experimental data.

Assay Name	Site 1 [L]	Site 2 [R]		Sequence
*RAG2*_1/*RAG2*_5_PCR	*RAG2*_1	*RAG2*_5	Primer F	GTGGTAAGAGATCAGGGTGAGTTC
			Primer R	GGTAAGAGATCAGGGTGAGTTCAG
TRANS1_ddPCR	*RAG1*_1	*RAG1*_7	Primer F	GTGTGAAAAGGAGGAAGAGG
			Primer R	CTTAGGGTGGGCTTTTAACAA
			Probe	CCTTGTCCAGGACTGCTGGAGA
TRANS5_ddPCR	*RAG2*_1	*RAG2*_5	Primer F	CTATGATCAGCACCTAACATG
			Primer R	GGATCCTGAAATAATAGGAGACAT
			Probe	CCATTCTCCTGCCTCAGCCTCC
TRANS9_ddPCR	*RAG2*_7	*RAG2*_1	Primer F	CCAGAACAGAGGCTAGAGAAT
			Primer R	GGCATGCACTGAAGTATTTC
			Probe	TGCAGAGATTGTGGGCGACAG
TRANS10_ddPCR	*RAG1*_2	*RAG1*_1	Primer F	TACTTGAAGGGGCTAAGGTGG
			Primer R	CCTCTTGCTTTCTCGTTGTC
			Probe	ATGGCTGAGTTGGGACTGGC
*EMX*_ddPCR1	*EMX1*_2	*EMX1*_1	Primer F	ACATAACGCTTTACCTTCACTT
			Primer R	ATGCATCAAAACAAAAGGGAGA
			Probe	TCCGAGGAGAAGGCCAAGTGG
*EMX*_ddPCR2	*EMX1*_3	*EMX1*_9	Primer F	CAGGAATAGCCCTACAAAGGT
			Primer R	AAGGGAGAATGAGAAAAGCTTC
			Probe	AAGCAAGCTTTTCCTGACGCC
*RAG2*_10/*RAG2*_7	*RAG2*_10	*RAG2*_7	Primer F	GGCATTAAGTAATCTAGACAGAGCA
			Primer R	GGCAAGCAGATGGTCATACACTC
			Probe	GAGCTCAAGTTCAGTGCTGTAGTGT
*CCRL2*_ddPCR	Control assay		Primer F	GCTGTATGAATCCAGGTCC
			Primer R	CCTCCTGGCTGAGAAAAAG
			Probe	TGTTTCCTCCAGGATAAGGCAGCTGT

Supplementary Table 3 **Primers list.** For each translocation event tested, an event-specific assay was designed with a forward primer for site 1[L], and a reverse primer for site 2 [R]. Specific probes were designed for each ddPCR assay. To ensure specificity, each primer and probe was confirmed to be complementary to only one site in the genome.

Supplementary Notes

Supplementary Note 1: **CRISPECTOR user manual – Installation, usage and detailed examples of the input files and the output report**

1. Installation

CRISPECTOR can be installed using the conda package manager Bioconda (for Linux & macOS), or it can be run using the Docker containerization system (for Linux & macOS).

1.1. Bioconda

To install CRISPECTOR using Bioconda, download and install Anaconda Python 3.7, following the instructions at: https://www.anaconda.com/distribution/.

Open a terminal and type:

```
conda config --add channels defaults
conda config --add channels bioconda
conda config --add channels conda-forge
```

To install CRISPECTOR into the current conda environment, type:

```
conda install crispector
```

If you don't already have an existing environment with Python 3.7, you can create one first (e.g. named crispector_env) with:

```
conda create -n crispector_env python=3.7
conda activate crispector_env
conda install crispector
```

Check that CRISPECTOR is installed using the help command:

```
crispector –help
```

1.2. Docker

CRISPECTOR can be used via the Docker containerization system. This system allows CRISPECTOR to run on your system without configuring and installing additional packages. To run CRISPECTOR, first download and install docker:

https://docs.docker.com/engine/installation/

Next, Docker must be configured to access your hard drive and to run with sufficient memory. These parameters can be found in the Docker settings menu. To allow Docker to access your

hard drive, select ‘Shared Drives’ and make sure your drive name is selected. To adjust the memory allocation, select the ‘Advanced’ tab and allocate at least 4G of memory.

To run CRISPECTOR, make sure Docker is running, then open a terminal (Linux), command prompt (macOS) or PowerShell (Windows 10) and download the Docker image with:

```
docker pull quay.io/biocontainers/crispector:1.0.2b9--py_0
```

Rename CRISPECTOR docker image name:

```
docker image tag quay.io/biocontainers/crispector:1.0.2b9--py_0
crispector_image:latest
```

When done, verify that CRISPECTOR is installed using the command (help screen should show up):

```
docker run -v ${PWD}:/DATA -w /DATA crispector_image crispector -h
```

The -v parameter mounts the current directory to be accessible by CRISPECTOR, and the -w parameter sets the CRISPECTOR working directory. As long as you are running the command from the directory containing your data, you should not change the Docker -v or -w parameters.

2. Usage

CRISPECTOR is designed to run on two comparative NGS experiments - Treatment and Mock (control). CRISPECTOR has two running modes:

- **Multiplex-PCR input** (default mode) - CRISPECTOR assigns reads from the FASTQ files to target amplicons, as a pre-processing step.
- **Singleplex-PCR input** - In this mode, the input can be multiple singleplex-PCR for loci of the same experiment. Note, in this mode, CRISPECTOR won’t detect translocations (translocation reads are only amplified in a multiplex-PCR reaction). Also, do not mix singleplex FASTQ files from different experiments. CRISPECTOR evaluates NHEJ editing activity with respect to the experiment background noise. Thus, mixing experiments will cause wrong background noise estimation.

Please note that in both modes, adapters need to be trimmed in a pre-processing step.

2.1. Multiplex-PCR input

CRISPECTOR requires three parameters:

1. **Treatment input sequences** in the form of FASTQ files. Given by the `-t_r1` and `-t_r2` arguments. If the input is already pair-end merged or is a single-end, then omit `-t_r2`. FASTQ files can be gzip-compressed.

2. **Mock input sequences** in the form of FASTQ files. Given by the `-m_r1` and `-m_r2` arguments. If the input is already pair-end merged, then omit `-m_r2`. FASTQ files can be gzip-compressed.

3. An **experiment config file** (given by the `-c` argument). The experiment description in a CSV (Comma Separated Values) format. Template can be found at https://github.com/YakhiniGroup/crispector/blob/master/example/experiment_config_template.csv. The table has 11 columns:

   – **SiteName** [REQUIRED] - an identifier for the reference locus.
   – **AmpliconReference** [REQUIRED] - amplicon sequence used for the experiment (5'->3').
   – **gRNA** [REQUIRED] - gRNA sequence for each locus site. sequence should be supplied without the PAM sequence and without insertions or deletions.
   – **OnTarget** [REQUIRED] - a boolean indicating if the site is on-target (True) or off-target (False).
   – **ForwardPrimer** [Optional] - forward primers as were used in the experiment. If not supplied, primers are inferred from the amplicon reference.
   – **ReversePrimer** [Optional] - reverse primers as were used in the experiment. If not supplied, primers are inferred from the amplicon reference.
   – **TxInput1Path** - Leave empty for experiment with multiplexed input.
   – **TxInput2Path** - Leave empty for experiment with multiplexed input.
   – **MockInput1Path** - Leave empty for experiment with multiplexed input.
   – **MockInput2Path** - Leave empty for experiment with multiplexed input.
   – **DonorReference** [Optional] - If experiment is designed with HDR, then insert the amplicon sequence in the on-target row. Note that editing HDR activity isn't evaluated by CRISPECTOR.

   Where data is not available, leave the cell empty. If an entire column is empty, leave the entire column empty.

Command:

```
crispector -t_r1 tx_R1.fq.gz -t_r2 tx_R2.fq.gz -m_r1 mock_R1.fq.gz -m_r2
mock_R2.fq.gz -c exp_config.csv
```

Example:
You can download data and configuration for *EMX1* experiment (performed with rhAmpSeq). The experiment was designed with one on-target site and 10 off-target sites.
The compressed experiment files can be found at https://github.com/YakhiniGroup/crispector/raw/master/example/EMX1_11_sites_50k_reads.zip, and it contains the following files:

- EMX1_config.csv
- EMX1_tx_R1.fq.gz
- EMX1_tx_R2.fq.gz

- EMX1_mock_R1.fq.gz
- EMX1_mock_R2.fq.gz

The FASTQ files contain the first 50,000 reads of the experiment. Full sequencing can be found in NCBI as SRA accession: PRJNA630002.

With Conda:

```
crispector -t_r1 EMX1_tx_R1.fq.gz -t_r2 EMX1_tx_R2.fq.gz -m_r1 EMX1_mock_R1.fq.gz
-m_r2 EMX1_mock_R2.fq.gz -c EMX1_config.csv
```

With Docker:

```
docker run -v ${PWD}:/DATA -w crispector_image crispector -t_r1 EMX1_tx_R1.fq.gz
-t_r2 EMX1_tx_R2.fq.gz -m_r1 EMX1_mock_R1.fq.gz -m_r2 EMX1_mock_R2.fq.gz -c
EMX1_config.csv
```

The above example data was tested on a MacBookPro (2.9 GHz Intel Core i7, 16 GB RAM) and on a Linux machine (3.3 GHz Intel Core i9, 64 GB RAM). CRISPECTOR runtime on a single thread is 110 seconds and 100 seconds, respectively. CRISPECTOR runtime on a single thread on the full experiment (*EMX1*, XT 2p in HEK-CAS9) is ~5 minutes on both computers.

2.2. Singleplex-PCR input

Usage with singleplex-PCR input (FASTQ file for each locus site) is identical to multiplex-PCR input with the following exception - Full paths to the FASTQ files are given in the **experiment config file** (`-c`), instead of the command-line arguments (`-t_r1`, `-t_r2`, `-m_r1` & `-m_r2`).
The FASTQ file paths should be supplied for each target locus in the following experiment configuration columns:

- **TxInput1Path** and **TxInput2Path** - Full paths of treatment input sequences in the form of FASTQ files. If the input is already pair-end merged or is a single-end, then leave the column **TxInput2Path** empty. FASTQ files can be gzip-compressed.
- **MockInput1Path** and **MockInput2Path** - Full paths of mock input sequences in the form of FASTQ files. If the input is already pair-end merged or is a single-end, then leave the column **MockInput2Path** empty. FASTQ files can be gzip-compressed.

All other columns of **experiment config file** (`-c`) should be specified as in multiplex-PCR input.

Command:

```
crispector -c exp_config.csv
```

Example:
You can download data and configuration for *EMX1* experiment (performed with rhAmpSeq). Experiment was designed with one on-target site and 10 off-target sites. The FASTQ files

contain the first 5,000 reads for each target locus. Loci Sites were binned into separated FASTQ files using bowtie2. The compressed experiment files can be found at https://github.com/YakhiniGroup/crispector/raw/master/example/EMX1_11_sites_singleplex_input_500k_reads.zip. Make sure you change "PATH_TO_DIRECTORY" in the "EMX1_config.csv" file to your local directory path.

With Conda:

```
crispector -c EMX1_config.csv
```

With Docker:

```
docker run -v ${PWD}:/DATA -w /DATA crispector_image crispector -c
EMX1_config.csv
```

The above example data was tested on a MacBookPro (2.9 GHz Intel Core i7, 16 GB RAM) and on a Linux machine (3.3 GHz Intel Core i9, 64 GB RAM). CRISPECTOR runtime on a single thread is 100 seconds and 90 seconds, respectively. CRISPECTOR runtime on a single thread on the full experiment (*EMX1*, XT 2p in HEK-CAS9) is ~5 minutes on both computers.

2.3. All optional parameters

Relevant for both running modes - Multiplex and singleplex input.

```
Usage: crispector [OPTIONS]

  Accurate estimation of off-target editing activity from comparative NGS data

Options:
  -t_r1, --tx_in1 PATH            Tx read 1 input path or Tx merged FASTQ file
  -t_r2, --tx_in2 PATH            Tx read 2 input path, if FASTQ files aren't
merged
                                  [OPTIONAL]
  -m_r1, --mock_in1 PATH          Mock read 1 input path or mock merged FASTQ
file
  -m_r2, --mock_in2 PATH          Mock read read 2 input path, if FASTQ files
aren't
                                  merged [OPTIONAL]
  -c, --experiment_config PATH    A CSV (Comma Separated Values) file with the
                                  experiment data. Table has 11 columns:
                                  SiteName, AmpliconReference, gRNA, OnTarget,
                                  ForwardPrimer,
                                  ReversePrimer,TxInput1Path TxInput2Path,
                                  MockInput1Path, MockInput2Path,
                                  DonorReference. The first 4 columns are
required,
                                  the rest are optional. Header
                                  should be specified by the above order. Please
                                  check the README on GitHub further
                                  details and examples.  [required]
```

```
  -o, --report_output PATH        Path to output folder
  --cut_site_position INTEGER     Expected cut-site position with respect to the
3'
                                  end of the provided sgRNA
                                  sequence. Note, the sgRNA sequence must be
entered
                                  without the PAM.  [default: -3]
  --crispector_config PATH        Path to crispector configuration in YAML
format.
                                  See "Advanced usage" section in
                                  README on GitHub for further.
  --fastp_options_string TEXT     Try "fastp --help" for more details
  --min_num_of_reads INTEGER      Minimum number of reads (per locus site) to
                                  evaluate edit events  [default: 500]
  --min_read_length_without_primers INTEGER
                                  Filter out any read shorter than
                                  min_read_length_without_primers + length of
forward
                                  and reverse primers. This threshold filters
primer-
                               dimmer effect reads.  [default: 10]
  --max_edit_distance_on_primers INTEGER
                                  Maximum edit distance to consider a read
                                  prefix (or suffix) as a match for a primer.
                                  [default: 8]
  --amplicon_min_score FLOAT RANGE
                                  Minimum normalized alignment score to consider
a
                               read alignment as valid. Normalized
                                  alignment score is defined as the Needleman-
Wunch
                                  alignment score divided by the
                                  maximum possible score. Below this alignment
                                  threshold, reads are discarded. [default: 30]
  --translocation_amplicon_min_score FLOAT RANGE
                                  Minimum alignment score to consider a read with
                                  primer inconsistency as a possible
                                  translocation. Should be higher than –
                                  amplicon_min_score, because translocations
                                  reads are noisier.Score is normalized between 0
                                   (not even one bp match) to 100 (read
                                  is identical to the reference) [default: 80]
  --min_editing_activity FLOAT RANGE
                                  Minimum editing activity (%). Sites with
editing
                                  activity lower than the minimum,
                                  will be discarded from the translocation
detection.
                                   [default: 0.1]
  --translocation_p_value FLOAT RANGE
                                  Translocations statistical significance level.
This
                                  threshold is applied on the
                                  corrected p_value,FDR (false discovery rate).
                                   [default: 0.05]
```

```
  --disable_translocations        Disable translocations detection  [default:
False]
  --override_noise_estimation     Override noise estimation with default q
parameter
                                  from crispector_config file. It's
                                  advisable to set this flag for experiment with
a
                                  low number of off-target sites
                                  (<5). q is defined as the probability of an
indel
                                  to occur through an edit event.
                                  Check CRISPECTOR paper for more details.
[default:
                                  False]
  --confidence_interval FLOAT RANGE
                                  Confidence interval for the evaluated editing
                                  activity  [default: 0.95]
  --enable_substitutions          Enable substitutions events for the
quantification
                                  of edit events  [default: False]
  --suppress_site_output          Do not create plots for sites (save memory and
                                  runtime)  [default: False]
  --keep_intermediate_files       Keep intermediate files for debug purposes
                                   [default: False; required]
  --verbose                       Higher verbosity  [default: False]
  -h, --help                          Show this message and exit.
```

2.4. Advanced usage - CRISPECTOR configuration file

Advanced users can further tune CRISPECTOR parameters using CRISPECTOR's configuration file. A path to an alternative configuartion file can be given to CRISPECTOR with `(--crispector_config <configuration_file_path>)`. The default configuration file (which can be copied and replaced with alternative values) can be found at:

https://github.com/YakhiniGroup/crispector/blob/master/crispector/config/default_config.yml.

Two main parameter types can be tuned in the configuration file:

4. Alignment
5. NHEJ inference

Alignment

Parameters for Needleman-Wunch algorithm:

- match_score (int): Match option for Needleman-Wunsch alignment (default: 5)
- mismatch_score [int]: Mismatch option for Needleman-Wunsch alignment (default: -4)
- open_gap_score [int]: Gap open option for Needleman-Wunsch alignment (default: -25)
- extend_gap_score [int]: Gap extend option for Needleman-Wunsch alignment (default: 0)

- substitution_matrix [string]: Replace mismatch_score option with a matrix score. Any substitution matrix name from BioPython (Bio.SubsMat.MatrixInfo) can be used. E.g. “blosum62”. (default: “”, meaning no substitution matrix)

NHEJ inference

Parameters for NHEJ inference. This option is advisable only for users that read CRISPECTOR paper:

- default_q [int] - The probability of an indel to occur through an edit event. Used when `--override_noise_estimation` is set. (default: -3).
- window_size [int] - Defines the size (in bp) of the window extending from the position of the expected cut-site. Outside this window, indels won’t be accounted as edits. Changing this field requries changing the *prior probability* for each indel type as well (default: 10)
- IndelType: An hierarchical bundle of parameters that defines the indel types and their *prior probabilities* (as defined in CRISPECTOR paper). For each indel type (Deletion, Insertions, Substitutions or Mixed) user can define the aggregation level for different indel lengths and the indel type *prior probability*. For example, consider the following:

```
Deletions:
    del_len_1:
        min: 1  # min indel length
        max: 1  # max indel length
        pos_prior: [0.0001, 0.0001, 0.0001, 0.0001, 0.0001, 0.0001, 0.0001,
                    0.001, 0.1, 0.5, 0.5, 0.1, 0.001, 0.0001, 0.0001, 0.0001,
                    0.0001, 0.0001, 0.0001, 0.0001]
```

The user defined indel type for deletion of length 1 (because only length 1 ∈ [`min`, `max`]). The user also defined the *prior probability* (`pos_prior`) for every bp in the window around the expected cut-site. The expected cut-site is located right to the middle index. In the above exmaple, window size is 10, therefore the expected cut-site is between index 10 and 11 (between `0.5`, `0.5`). The *prior probability* is symmetrical around the expected cut-site. Thus, the length of the `pos_prior` list is 2*`window_size`, except for `Insertions` where it’s 2*`window_size` + 1. That’s because Insertions are defined between reference sequence bases, and not “on” them. Please note that `del_len_1` is just the name of the indel type, and it doesn’t affect anything else.

3. CRISPECTOR output - HTML-based report

CRISPECTOR generates an HTML-based report to support user interpretation and further analysis of the outcomes. A compressed output directory example can be found at https://github.com/YakhiniGroup/crispector/raw/master/example/EMX1_11_sites_50k_reads_output.zip. Once downloaded, apply “Extract all” or “Unzip here” with a zip application (such as Winzip) and open “report.html”. The report is best viewed through Google Chrome.

The report contains plots and tables that summarize the experiment results. For example, multiple off-target editing activity including statistical confidence indicators and translocation results. The

report also contains read statistics, such as the number of assigned and aligned reads in each processing step in the different loci. In addition, plots are generated for each individual locus: distribution of edit events, classifier results for each modification type and reference position, and alignments of all edited reads, in a graphical format. Furthermore, all aligned reads in treatment and mock, whether assigned to a specific locus or determined as primer inconsistent (and therefore maybe representing a translocation event), are saved in a set of CSV files.

Supplementary Note 2: **Human validated dataset for the task of active off-target site classification**

The dataset:
Out of all 1,161 instances in our experimental data (see Methods section), real editing activity was estimated by human validation for 19 sites (see Supplementary Note 4). These 19 sites have disagreement between CRISPECTOR and CRISPResso2 in the active site classification task. Namely, CRISPECTOR classified a site as active (determined by estimated editing activity $\geq 0.1\%$) and CRISPResso2 as inactive (estimated editing activity $\leq 0.1\%$), or vice-versa. Description of these 19 experiments with the validated real editing activity, as well as CRISPECTOR, CRISPResso2 and ampliCan results can be found in Supplementary Data 1 file: "human_validated_dataset_results.xlsx".

These aligned and filtered reads that were used to validate the real editing activity of the 19 sites can be found in the Supplementary file:
"S3_aligned_and_filtered_reads_for_human_validated_dataset.zip".
Each site has its own directory with 4 files: Tx aligned read table (CSV format), Mock aligned read table, Tx filtered reads (FASTA format), and Mock filtered reads.

Human examination process:
The editing activity rate for all sites with disagreement between CRISPResso2 and CRISPECTOR was human validated. These sites had a relatively small number of reads with indels around the expected cut-site (due to low editing activity), which made the human validation process feasible. Each read sequence with insertion or deletion, 10bp on either side of the cut site was examined. The disagreement between the two tools was called when two conditions were met. First, one tool determined the off-target site as active (editing rate $\geq 0.1\%$) and the other as inactive (editing rate $\leq 0.1\%$). Second, the relative difference is higher than 25%. Relative difference is defined as $\frac{|p-q|}{q}$, Where p and q are the estimated editing activities, with q being the smaller.

In order to assess the real editing rate, read alignments were verified visually, by two independent scientists, and compared to mock reads. Moreover, filtered reads, due to a low alignment score, were also examined. All unassigned reads from the demultiplexing step were searched for the best site assignment with respect to the optimal normalized Needleman-Wunch alignment score and were validated as well. While alignment tools can detect different alignments and miss real editing, human validation can visually inspect all the various options around the cut site, which for SpCas9 is 3bp upstream to the cut site from the 3' end of the gRNA. This includes assessing alternative PAM sequence relative to its cut site, sequence errors caused by a homo-dimer sequence, and assessing all the possible alternative alignments (consider examples below and Supplementary Note 3).

In each human evaluation, any sequence that was suspected as an editing event by CRISPECTOR was aligned to a reference sequence of the specific off-target site, using Clustal Omega multiple sequence alignment program (https://www.ebi.ac.uk/Tools/msa/clustalo). We confirmed that the editing event overlaps the cut site (3bp upstream to the PAM sequence). Next, we verified that the indel event is absent from the Mock sample. If the editing event was located near the cut site, but

not overlapping it, then we looked for an alternative alignment option that can match an alternative PAM. Through this process, we verified CRISPECTOR editing calls. Furthermore, this step was taken to make sure that reads were not overlooked due to the primer-matching algorithm that is described in Supplementary Note 7. Finally, sites with a low number of reads ($\leq$ 500) were discarded from the evaluation process.

Guided example:

Consider the following alignment reads for off-target site 51 in *RAG1 XT 2p*, *HEK-CAS9* experiment, as an example for human validation. In this site, the PAM sequence is GGG and at the expected cut site located a homodimer sequence GGG. We note that in this region an alternative PAM may be active. During the human validation process, we want to take into account possible sequencing errors[4].

Consider the treatment reads aligned and compared to the mock reads. We distinguish five types of reads represent indels around the expected cut-site:

1. 1bp deletion located at the cut site: 5 events in the treatment sample versus 0 events in the mock. Original PAM marked in Red. Alternative PAM sequence is marked in Blue.

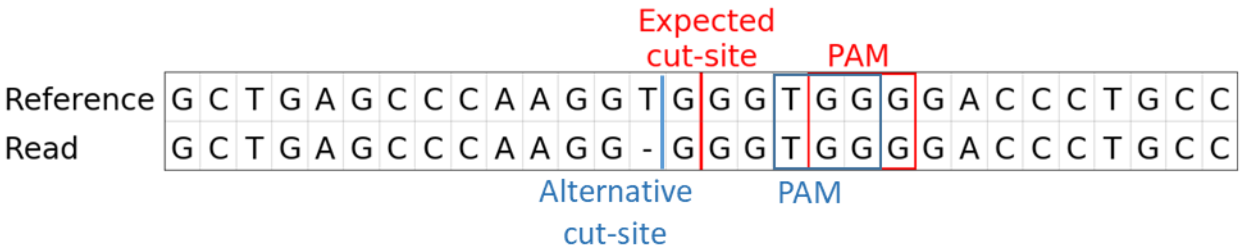

CRISPECTOR calls these reads as edited.
CRISPResso2 considers these reads as unedited because the 1bp deletion event is not at the expected cut-site.
Human evaluation calls these reads as edited due to the alternative PAM sequence TGG.

2. 1bp deletion located at the cut site: 17 events in the treatment sample versus 41 events in the mock.

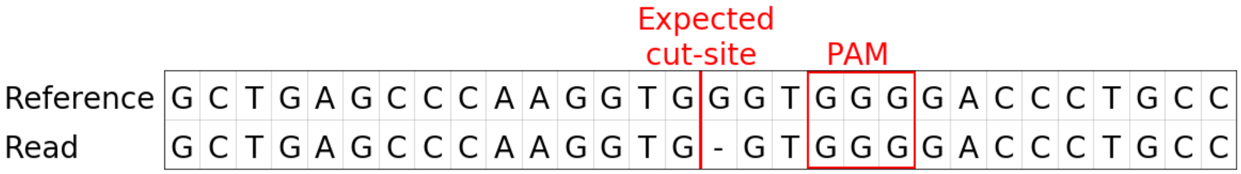

CRISPECTOR calls these reads as unedited.
CRISPResso2 - These reads negative contributed to the final editing activity estimation, due to the treatment minus mock subtraction approach. Specifically, the contribution is: 0.058% - 0.076%=-0.018%
Human evaluation calls these reads as un-edited due to proportions of these reads in the treatment (0.058%) and in the mock (0.077%), indicating that this is a sequence error.

3. 4bp deletion located at the cut site: 18 events in the treatment sample versus 3 events in the mock.

Expected cut-site PAM

Reference	G	C	T	G	A	G	C	C	C	A	A	G	G	T	G	G	G	T	G	G	G	G	A	C	C	C	T	G	C	C
Read	G	C	T	G	A	G	C	C	C	A	A	G	G	T	G	-	-	-	-	G	G	G	A	C	C	C	T	G	C	C

CRISPECTOR calls these reads as edited.
CRISPResso2 - These reads contributed to the final editing activity estimation 0.061% - 0.06% = 0.055%
Human evaluation calls these reads as edited due to the indel position at the expected cut-site and low mock activity (0.005%) compared to the treatment (0.062%).

4. 4bp deletion located at the cut site: 1 event in the treatment sample versus 0 events in the mock.

Expected cut-site PAM

Reference	G	C	T	G	A	G	C	C	C	A	A	G	G	T	G	G	G	T	G	G	G	G	A	C	C	C	T	G	C	C
Read	G	C	T	G	A	G	C	C	C	A	-	-	-	-	G	G	G	T	G	G	G	G	A	C	C	C	T	G	C	C

CRISPECTOR calls this read as unedited. This read was missed due to a low frequency of this indel type and position, which is not at the expected cut-site.
CRISPResso2 considers this read as unedited because the 4bp deletion event is not at the expected cut-site.
Human evaluation calls these reads as edited due to the alternative PAM sequence TGG.

5. 17bp deletion located around the cut site: 9 events in the treatment sample versus 0 events in the mock.

Expected cut-site PAM

Reference	G	C	T	G	A	G	C	C	C	A	A	G	G	T	G	G	G	T	G	G	G	G	A	C	C	C	T	G	C	C
Read	G	C	T	G	A	G	C	C	C	A	A	G	G	T	G	-	-	-	-	-	-	-	-	-	-	-	-	-	-	-

CRISPECTOR calls these reads as edited.
CRISPResso2 calls these reads as edited.
Human evaluation calls these reads as edited.

CRISPECTOR final editing activity estimation is 0.11% (CI ± 0.038%).
CRISPResso2 final editing activity estimation is 0.067%.
Human evaluation final editing activity is 0.113%.

When running ampliCan on this example, the ampliCan final report generation crashes. We also note, the ampliCan doesn't provide read level activity calling information to the user.

Supplementary Note 3: **optimal read alignments can place real edited modifications outside the expected cut-site**

Even with biologically informed alignment algorithms, optimal read alignments are not always the best possible explanation for the biological mechanism that generated the read. Consider the following 19 unique to treatment reads (off-target site 6 of *RAG1*, sg, 1:2.5 no EE, 4uM in CD34+):

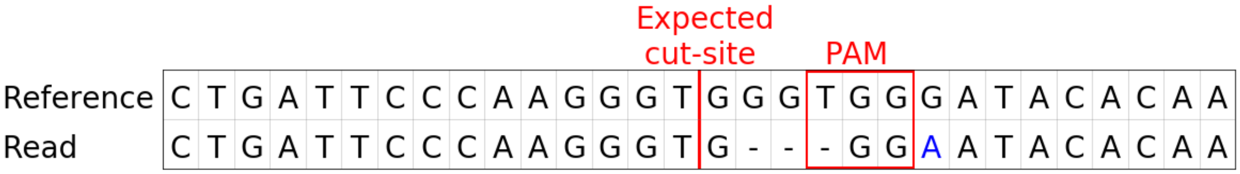

Another possible alignment, although non-optimal with respect to the alignment score is:

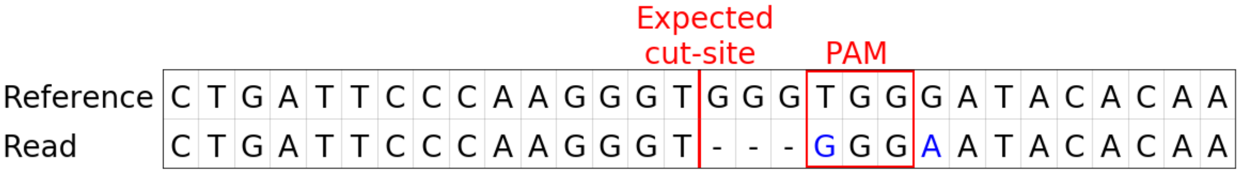

The alternative alignment introduces a deletion of length 3 on the expected cut-site, but with an addition of one mismatch. It turns out, that the first base of the PAM (T) is a noisy position, probably due to sequencing artifacts, with a mismatch rate of 0.81 % (712 mismatches out from 82,599 reads). Thus, optimal alignments can place real edited modifications outside the expected cut-site. Therefore, the ability to detect alternative cut-sites is crucial.

Supplementary Note 4: **CRISPECTOR detects editing activity due to the existence of an alternative PAM sequence**

Accurate editing activity estimation also depends on the detection of alternative cut-sites. One possible source is the existence of an alternative PAM sequence. The detection of alternative cut-sites was observed on a few different genomic loci in our human validated experimental data. For example, consider off-target site 5 in an *HPRT* experiment (Supplementary Figure 6a). CRISPECTOR classified as edited 28 reads with insertions one base left to the expected cut-site, with a compatible PAM sequence (GGG). Note that the original PAM sequence is GGA and that the 20 bp sequence corresponding to the original PAM has higher similarity (smaller edit distance) to the sgRNA used, as compared to the 20 bp sequence that corresponds to the alternative PAM.

Another example is off-target site 61 in a *RAG1* experiment (Supplementary Figure 6b). CRISPECTOR classified as edited 48 reads with deletions of length 2 five bases left to the expected cut-site, with a compatible PAM sequence (TGG). Note that CRISPECTOR classified these 48 reads despite that the mock experiment had 4 reads with the same indel type and position. The probability of seeing the observation of Tx=48 and M=4 under the null model of equally distributed indels is $5.53 * 10^{-14}$ (Hypergeometric tail probability, as described in the methods section). Moreover, site 61 is an active off-target site (editing activity $\geq$ 0.1%, even without these 48 reads) with a high-depth (number of reads higher than 80K in both sites). The above observations strengthen the claim that this site, indeed, has an alternative cut-site due to the existence of an alternative PAM sequence.

The reads that support the above two examples were individually validated by human inspection and are fully reported in Supplementary Note 2.

Supplementary Note 5: **Tool running parameters**

CRISPECTOR – We ran CRISPECTOR with the default parameters, as described in the user manual at https://github.com/YakhiniGroup/crispector.

CRISPResso2 – We ran CRISPRessoPooled with the default parameters separately on Tx and M experiments. Final editing activity was determined as $\max(0, \widehat{p_{Tx}} - \widehat{p_M})$, where $\widehat{p_{Tx}}$ and $\widehat{p_M}$ denote CRISPResso2 estimated NHEJ activity in Tx and M, respectively. When using CRISPResso merging software, FLASH[5], many off-target sites had no read assignment. Thus, we pre-processed and merged the pair-end reads with PEAR software.

ampliCan - We ran ampliCan 1.8.2 with five different operation modes, alternating qualification window size and minimum indel frequency parameters. For fair comparison, we searched for a configuration with the lowest number of sites with disagreement between ampliCan and other tools in the active off-target site classification task:

Parameters	Number of sites with disagreement between CRISPECTOR & ampliCan	Number of sites with disagreement between CRISPResso2 & ampliCan
Default parameters (cut_buffer=5, min_freq=1)	477	481
ampliCan Conservative parameters (cut_buffer=5, min_freq=15)	477	481
cut_buffer=5, min_freq=0	152	152
cut_buffer=1, min_freq=1	282	284
cut_buffer=1, min_freq=0	142	136

All ampliCan results presented in this paper are based on the last run.

Supplementary Note 6: **Concordance of activity rates in *EMX1* experiments**

We investigated the concordance of our positive indel off target activity calls in *EMX1* (see Methods). This includes four paired experiments (with different activity levels, as reflected in the on-target rates). We focus on sites where an activity of $\geq 0.1\%$ is called:

- *EMX1 IVT* pair:
  EMX1_IVT_1_1.2_no_EE_4uM_CD34Plus – The low editing activity experiment.
  EMX1_IVT_1_1.2_EE_4uM_CD34Plus – The high editing activity experiment.
  CRISPECTOR - 1 out of 2 sites are concordant.
  CRISPResso2 -1 out of 2 sites are concordant.

- *EMX1 XT_2p* pair:
  EMX1_XT_2p_1_1.2_no_EE_4uM_CD34Plus – The low editing activity experiment.
  EMX1_XT_2p_1_1.2_EE_4uM_CD34Plus – The high editing activity experiment.
  CRISPECTOR - 4 out of 4 sites are concordant.
  CRISPResso2 - 4 out of 4 sites are concordant.

- *EMX1 crRNA* pair:
  EMX1_crRNA_2p_1_1.2_no_EE_4uM_CD34Plus – The low editing activity experiment.
  EMX1_crRNA_2p_1_1.2_EE_4uM_CD34Plus – The high editing activity experiment.
  CRISPECTOR - 3 out of 4 sites are concordant.
  CRISPResso2 - 4 out of 5 sites are concordant.

- *EMX1 sg* pair:
  EMX1_sg_1_1.2_no_EE_4uM_CD34Plus – The low editing activity experiment.
  EMX1_sg_1_1.2_EE_4uM_CD34Plus – The high editing activity experiment.
  CRISPECTOR - 4 out of 4 sites are concordant.
  CRISPResso2 - 3 out of 3 sites are concordant.

- *RAG1* pair:
  RAG1_XT_2p_1_2.5_EE_4uM_HEK293 – The low editing activity experiment.
  RAG1_XT_2p_HEKCas9 – The high editing activity experiment.
  CRISPECTOR - 46 out of 47 sites are concordant.
  CRISPResso2 - 44 out of 45 sites are concordant.

- *RAG2* pair:
  RAG2_XT_2p_1_2.5_EE_4uM_HEK293 – The low editing activity experiment.
  RAG2_XT_2p_HEKCas9 – The high editing activity experiment.
  CRISPECTOR - 27 out of 27 sites are concordant.
  CRISPResso2 - 28 out of 28 sites are concordant.

CRISPECTOR - In total we observed 85 out of 88 pairs to be concordant, leading to a p-value of 1.11*10^-16 (tail probability of a $Binom(n = 88, p = 0.5)$ distribution).
CRISPResso2 - In total we observed 84 out of 87 pairs to be concordant, leading to a p-value of 1.11*10^-16.

All *EMX1* results used for the analysis are listed in the tables below, for both CRISPECTOR and CRISPResso2 (Green – concordant instances, red – discordant instances, other – inactive instances with estimated editing activity $\leq 0.1\%$).

CRISPECTOR:

Site Name	*IVT*		*XT_2p*		*crRNA*		*sg*	
	Low	High	Low	High	Low	High	Low	High
On-target	26.33	31.08	9.40	73.31	13.55	82.61	20.52	79.60
EMX1_2	0.01	0.24	0.87	1.22	0.09	0.93	0.70	0.75
EMX1_3	0.40	0.16	1.95	18.18	0.43	6.35	2.73	19.79
EMX1_4	0.00	0.06	0.08	0.00	0.00	0.00	0.00	0.00
EMX1_5	0.00	0.00	0.00	0.00	0.00	0.05	0.02	0.02
EMX1_6	0.00	0.07	0.07	0.27	0.00	0.06	0.03	0.12
EMX1_7	0.00	0.00	0.00	0.00	0.00	0.13	0.00	0.04
EMX1_8	0.02	0.05	0.00	0.03	0.01	0.02	0.00	0.00
EMX1_9	0.00	0.00	0.03	0.02	0.11	0.05	0.03	0.00
EMX1_10	0.08	0.00	0.02	0.31	0.00	0.03	0.10	0.15

CRISPResso2:

Site Name	*IVT*		*XT_2p*		*crRNA*		*sg*	
	Low	High	Low	High	Low	High	Low	High
On-target	23.40	27.21	7.40	68.83	11.04	78.00	17.57	74.87
EMX1_2	0.09	0.19	0.25	1.22	0.05	0.94	0.15	0.92
EMX1_3	0.19	0.03	1.59	17.39	0.24	6.07	2.48	19.11
EMX1_4	0.00	0.00	0.07	0.00	0.00	0.00	0.00	0.00
EMX1_5	0.00	0.00	0.00	0.00	0.00	0.10	0.00	0.00
EMX1_6	0.00	0.07	0.06	0.32	0.00	0.03	0.03	0.12
EMX1_7	0.00	0.00	0.00	0.00	0.10	0.05	0.00	0.04
EMX1_8	0.00	0.03	0.00	0.04	0.00	0.13	0.00	0.00
EMX1_9	0.00	0.00	0.01	0.01	0.03	0.02	0.03	0.00
EMX1_10	0.02	0.00	0.00	0.30	0.00	0.05	0.04	0.09

Supplementary Note 7: **CRISPECTOR demultiplexes reads using primer-matching algorithm**

CRISPECTOR assigns reads to loci by finding, for the read prefix and suffix, the forward and reverse primers that best match it, from an edit distance perspective. Candidate loci are represented by a list of primers. A match is considered successful for a Levenshtein edit distance smaller than a configurable threshold (in our experiments, maximum acceptable edit distance is set to 8). NGS noise levels should be significantly lower than the maximum edit distance on primers, and indel editing activity should not occur on primers (otherwise they would not be amplified). Thus, reads with at least one unmatched primer were filtered out. In order to prevent read filtering due to wrong amplicons provided by the user or by some other phenomena, CRISPECTOR produces a user warning when identical high-frequency reads are filtered out. All unassigned reads are provided to the user in a FASTA file format.

Reads with an inconsistent assignment, e.g. forward primer belongs to loci λ_1 and reverse primer belongs to loci λ_2, are searched for an optimal alignment. Each read is aligned to three different sequences: λ_1 reference, λ_2 reference, and the putative translocation reference for the pair (λ_1, λ_2). Translocation reference is constructed from the concatenation of the first half of λ_1 reference (up to expected cut-site) and the second half of λ_2 reference (from to expected cut-site), with respect to the primer directions. Alignment scores are normalized by the reference lengths. The highest score assigns the read, whether as putative translocation or as matching to λ_1 or λ_2.

Supplementary Note 8: **CRISPECTOR aligns reads with an optimized version of the Needleman-Wunch algorithm**

Most common alignment algorithms are not suitable for genome editing analysis because they lack the biological insights of the CRISPR-CAS9 mechanism. Insertions and deletions, short as well as long, are caused by nuclease cleavage events. This problem was addressed in previous works[6,7]. Therefore, CRISPECTOR uses Biopython[8] implementation of the Needleman-Wunch algorithm with optimized parameters for the detection of long indels (from [7], gap opening $= -25$, gap extension $= 0$, match $= 5$, mismatch $= -4$ and no end gap penalty). Moreover, similarly to [6], indels are "pushed" into the expected cut-site, without degrading the alignment score, which is optimal under the given cost function.

In the second step, low-quality alignments are filtered out. Low-quality alignments can be a result of sequencing artifacts, primer-dimer effects, and erroneously assignments. Erroneous reads are considered for filtering if their normalized alignment scores are lower than a configurable threshold (30% in our demonstration data). Normalized scores are calculated by dividing the alignment score by the site's maximum observed score. Finally, reads with low normalized scores are filtered only if their alignment representation in CIGAR (Compact Idiosyncratic Gapped Alignment Report) path length is larger than 8. CIGAR path length is only dependent in the number of indels in the read, and not in the indel types and lengths. The last step was taken in order to keep reads with extremely long deletions, which can lead to a low normalized alignment score. Reads that resulted from the primer-dimer effect are filtered out with a configurable minimum read length threshold.
All filtered reads are provided to the user in a FASTA file format.

Supplementary Note 9**: Indel types to be considering for classification**

In our experiments we defined and worked with the following 19 indel types:

1. Deletion of length 1
2. Deletion of length 2
3. Deletion of length 3
4. Deletion of length 4
5. Deletion of length 5
6. Deletion of length ≥ 6
7. Insertions of length 1
8. Insertions of length 2
9. Insertions of length 3
10. Insertions of length 4
11. Insertions of length 5
12. Insertions of length ≥ 6
13. Mixed of length 1*
14. Mixed of length 2*
15. Mixed of length 3*
16. Mixed of length 4*
17. Mixed of length 5*
18. Mixed of length ≥ 6*
19. Substitutions**

* mixed type is an indel compound from two or more consecutive indels.
** Substitutions are by default disabled from the analysis.

The above indel types are fully configurable. The aggregation of indels larger than length of 6 was found to produce accurate results on our demonstrated data, with no false-positive or false-negative due to the aggregation.

Supplementary Note 10: **Inferring q, the probability of an indel to occur through an edit event**

q estimation for each locus site is computed from the indels in the experiment. Utilizing the observation that almost all on-target treatment indels on the cut-site should originate from edit events, we can infer q. Denote $edit_{on}$ as the number of on-target Tx indels on the cut-site, the inferred edited indels. Respectively, denote $noise_{on}$ as the number of on-target M indels on the cut-site, the inferred background noise. Thus, by dividing $edit_{on}$ by the total number of indels on the cut-site, we can estimate q:

$$(S1)\quad q = \frac{edit_{on}}{edit_{on} + noise_{on}}$$

However, the above estimation has two shortcomings. First, $noise_{on}$, a single observation, is not a good approximation for the experiment's background noise. Second, many off-target sites have an unbalanced number of reads (Tx vs. M), that could cause false classification. In order to address these shortcomings, $\hat{\alpha}$, a background noise estimation, and a scaling factor (based on the number of reads) were incorporated into q estimation:

$$(S2)\quad q(j) = \frac{edit_{on}}{edit_{on} + \hat{\alpha} * N_{on} * \frac{N_M(j)}{N_{Tx}(j)}}, \qquad j \in \{1..num\ of\ sites\}$$

Where $N_{Tx}(j)$ and $N_M(j)$, are the Tx and M number of reads for locus site λ_j, and N_{on} is the total number of reads of the on-target mock experiment.

In order to estimate the background noise, $\hat{\alpha}$, we considered the observation of the mock indel frequency in each amplicon reference position of the site locus, $\alpha(j,i) = \frac{n_M(j,i)}{N_M(j)}$. Where $n_m(j,i)$ is the number of mock indels in locus site λ_j and reference position i. First, $\hat{\alpha}(j)$, the background noise estimation for locus site λ_j, is estimated as the 95th percentile of $\alpha(j,i)$. And second, $\hat{\alpha}$ is computed as the 95th percentile of $\hat{\alpha}(j)$. The selection of the 95th percentile, instead of median or average, is done to minimize false classification in high background noise experiments.

$q(j)$ is separately estimated for insertions, deletions and substitutions. The final $q(j)$ is averaged with a user-configurable pre-defined probability (0.9 in our experiments). This final step further reduces q(j) to encourage more conservative estimates (lower FN).

Supplementary Note 11**: Accounting for the effect of PCR duplication in Site 51 of *RAG1*, XT 2p in HEK293-Cas9**

Current approach – No PCR duplication filtering:

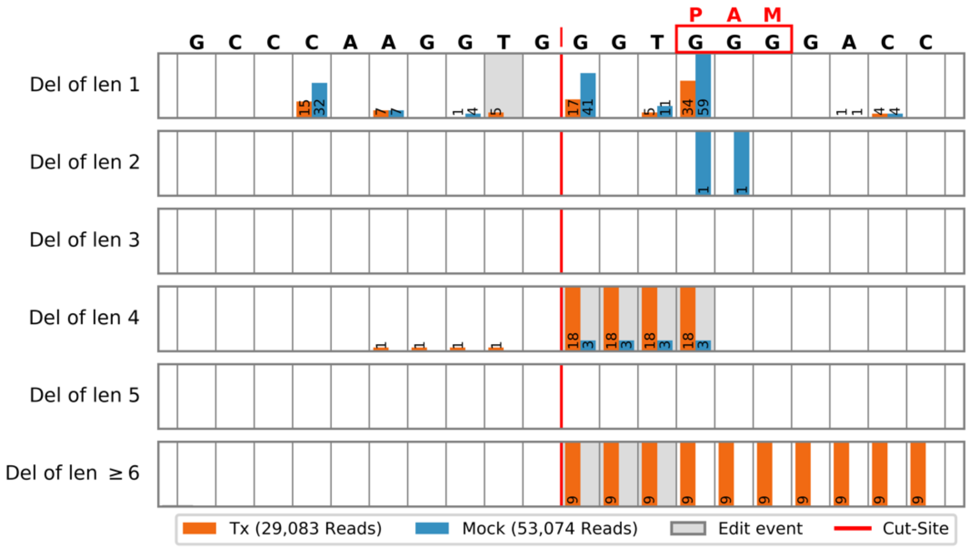

Final editing activity – 0.11%.

Conservative approach:

Given the assumption that any repetitive read in the mock experiment (except the amplicon reference read) is originated by a PCR duplication, we removed all identical reads from the mock. This approach can lead to FP estimation.

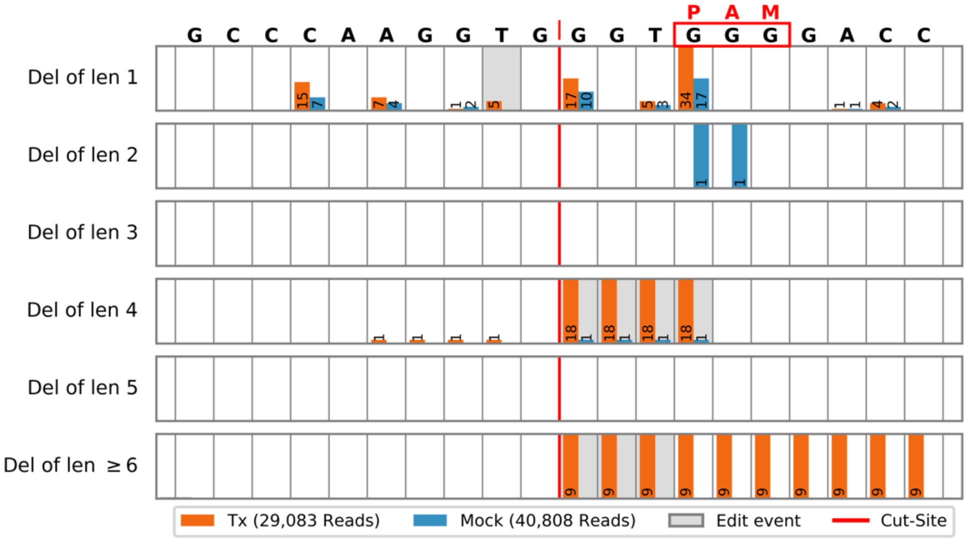

Final editing activity – 0.11%.

Although the final editing activity hasn't changed, note that in noisy positions, such as in the 1bp deletion at the cut-site, the mock indel frequency is now lower than the treatment indel frequency.

Anti-conservative approach:
Given the assumption that any repetitive read (except the amplicon reference read) is originated by a PCR duplication, we removed all identical reads form both the treatment and the mock experiments.

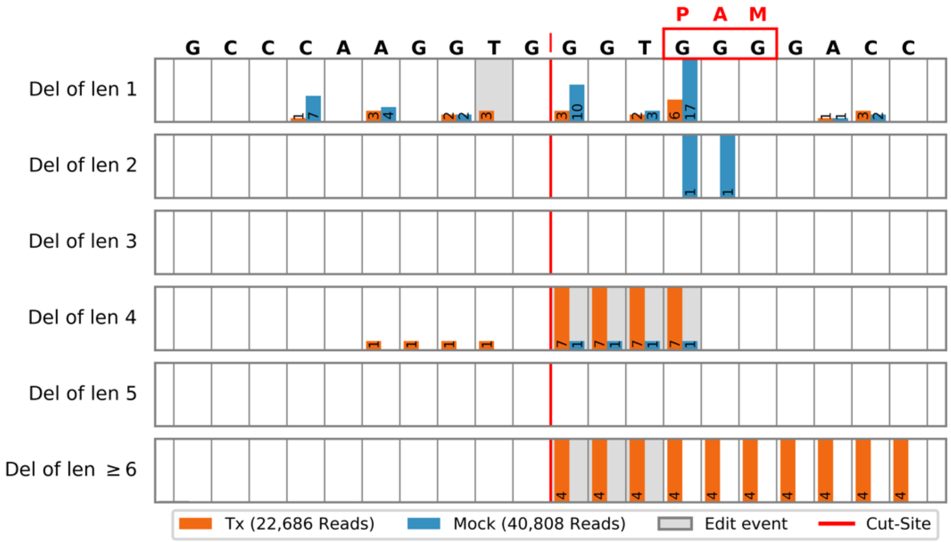

Final editing activity – 0.061%.

With the PCR duplication filtering for both the treatment and the mock, the editing activity estimation decreased. The activity decreased due to the background (total read) estimation. Most reads belong to the amplicon reference sequence. There is no simple or obvious way to evaluate the PCR duplication for these reads. As a result, the number of edited indels, as called by CRISPECTOR, notably decreased, while the total number of reads only slightly decreased.

References

1. Shapiro, J. *et al.* Increasing CRISPR efficiency and measuring its specificity in hematopoietic stem and progenitor cells using a clinically relevant system. *Mol. Ther. - Methods Clin. Dev.* (2020) doi:10.1016/j.omtm.2020.04.027.
2. Tsai, S. Q. *et al.* GUIDE-seq enables genome-wide profiling of off-target cleavage by CRISPR-Cas nucleases. *Nat. Biotechnol.* **33**, 187–198 (2015).
3. Myles Hollander; Douglas A. Wolfe; Eric Chicken. Nonparametric Statistical Methods. in (2014).
4. Médigue, C., Rose, M., Viari, A. & Danchin, A. Detecting and analyzing DNA sequencing errors: Toward a higher quality of the Bacillus subtilis genome sequence. *Genome Res.* **9**, 1116–1127 (1999).
5. Magoc, T. & Salzberg, S. L. FLASH: fast length adjustment of short reads to improve genome assemblies. *Bioinformatics* **27**, 2957–2963 (2011).
6. Clement, K. *et al.* CRISPResso2 provides accurate and rapid genome editing sequence analysis. *Nature Biotechnology* vol. 37 224–226 (2019).
7. Labun, K. *et al.* Accurate analysis of genuine CRISPR editing events with ampliCan. *Genome Res.* **29**, 843–847 (2019).
8. Cock, P. J. A. *et al.* Biopython: freely available Python tools for computational molecular biology and bioinformatics. *Bioinformatics* **25**, 1422–1423 (2009).
