# Supplementary material for: CRISPECTOR provides accurate estimation of genome editing translocation and off-target activity from comparative NGS data
Source: Nat Commun. 2021 May 24;12:3042. doi: 10.1038/s41467-021-22417-4 (PMC8144550; doi:10.1038/s41467-021-22417-4)
Supplement: Supplementary file 2 — Description of Additional Supplementary Files [file 41467_2021_22417_MOESM2_ESM.pdf]

Description of Additional Supplementary Files

File Name: Supplementary Data 1.

Description: Human validated dataset results – Supplementary Human validated off-target sites dataset as described in Supplementary Note S2.

File Name: Supplementary Data 2.

Description: Aligned and filtered reads for Human validated dataset – All aligned and filtered reads that were used to validate the real editing activity in the Human validated dataset, as described in Supplementary Note S2.
