# Supplementary material for: CRISPECTOR provides accurate estimation of genome editing translocation and off-target activity from comparative NGS data
Source: Nat Commun. 2021 May 24;12:3042. doi: 10.1038/s41467-021-22417-4 (PMC8144550; doi:10.1038/s41467-021-22417-4)
Supplement: Supplementary file 5 — Reporting Summary [file 41467_2021_22417_MOESM5_ESM.pdf]

nature research

Corresponding author(s): Ayal Hendel and Zohar Yakhini

Last updated by author(s): Feb 28, 2021

Reporting Summary

Nature Research wishes to improve the reproducibility of the work that we publish. This form provides structure for consistency and transparency in reporting. For further information on Nature Research policies, see our Editorial Policies and the Editorial Policy Checklist.

Statistics

For all statistical analyses, confirm that the following items are present in the figure legend, table legend, main text, or Methods section.

n/a	Confirmed	
☐	☒	The exact sample size (*n*) for each experimental group/condition, given as a discrete number and unit of measurement
☐	☒	A statement on whether measurements were taken from distinct samples or whether the same sample was measured repeatedly
☐	☒	The statistical test(s) used AND whether they are one- or two-sided<br>*Only common tests should be described solely by name; describe more complex techniques in the Methods section.*
☐	☒	A description of all covariates tested
☐	☒	A description of any assumptions or corrections, such as tests of normality and adjustment for multiple comparisons
☐	☒	A full description of the statistical parameters including central tendency (e.g. means) or other basic estimates (e.g. regression coefficient) AND variation (e.g. standard deviation) or associated estimates of uncertainty (e.g. confidence intervals)
☐	☒	For null hypothesis testing, the test statistic (e.g. *F*, *t*, *r*) with confidence intervals, effect sizes, degrees of freedom and *P* value noted<br>*Give P values as exact values whenever suitable.*
☐	☒	For Bayesian analysis, information on the choice of priors and Markov chain Monte Carlo settings
☐	☒	For hierarchical and complex designs, identification of the appropriate level for tests and full reporting of outcomes
☐	☒	Estimates of effect sizes (e.g. Cohen's *d*, Pearson's *r*), indicating how they were calculated

Our web collection on statistics for biologists contains articles on many of the points above.

Software and code

Policy information about availability of computer code

Data collection	No software was used for data collection.
Data analysis	Code availability (for CRISPECTOR SW):<br>CRISPECTOR command line tool is available as a Bioconda package and as a Docker image at http://bioconda.github.io/recipes/crispector/README.html. The source code and the installation and user manual are available online at https://github.com/YakhiniGroup/crispector.<br><br>Other software used for data analysis:<br>CRISPResso2 - https://github.com/pinellolab/CRISPResso2<br>FLASH - Magoc, T. & Salzberg, S. L. FLASH: fast length adjustment of short reads to improve genome assemblies. Bioinformatics 27, 2957–2963 (2011).<br>ampliCan - version 1.8.2 - https://www.bioconductor.org/packages/release/bioc/html/amplican.html<br>QuantaSoft Software, version 1.7, Regulatory Edition, for use with all QX100™ and QX200™ Droplet Digital™ PCR (ddPCR™) Systems

For manuscripts utilizing custom algorithms or software that are central to the research but not yet described in published literature, software must be made available to editors and reviewers. We strongly encourage code deposition in a community repository (e.g. GitHub). See the Nature Research guidelines for submitting code & software for further information.

Data

Policy information about availability of data

All manuscripts must include a data availability statement. This statement should provide the following information, where applicable:

- Accession codes, unique identifiers, or web links for publicly available datasets
- A list of figures that have associated raw data
- A description of any restrictions on data availability

The main data supporting the findings of this study are available within the paper and its Supplementary Information files. All sequencing data generated in this study have been deposited in the National Center for Biotechnology Information (NCBI) under SRA accession: PRJNA630002. The sequencing data from Shapiro et al., 2020, was obtained from the NCBI under SRA accession: PRJNA628100. The sequencing data from Vakulskas et al., 2018, was obtained from the NCBI under SRA accession: SRP150376. The raw data underlying Figure 3c as well as Supplementary Figure 11, are provided as a source data file. All other relevant data are available from the corresponding author upon request.

Field-specific reporting

Please select the one below that is the best fit for your research. If you are not sure, read the appropriate sections before making your selection.

☒ Life sciences ☐ Behavioural & social sciences ☐ Ecological, evolutionary & environmental sciences

For a reference copy of the document with all sections, see nature.com/documents/nr-reporting-summary-flat.pdf

Life sciences study design

All studies must disclose on these points even when the disclosure is negative.

Sample size	Five different gRNA targets were evaluated, including data taken from previously published studies. Some of the results were also established with different versions of modified gRNAs and/or Cas9 protein. Each gRNA has a different number of potential off-target sites. A total of 226 potential off-target sites were analyzed in this study. Exact p-value calculations are provided, using different test approaches, adjusted to the sample size and configuration.
Data exclusions	There was no data exclusion in this study.
Replication	For the HEK-293 experiments, three replicates were conducted. For the mPB CD34+ HSPCs experiments, three independently biological experiments were performed. All replications were successful.
Randomization	The gRNAs that were examined in this study target different on-target and off-target sites in the genome. Actual samples were partitioned in-vitro to yield balanced Tx and mock samples. This balance is, indeed, reflected in the NGS read numbers.
Blinding	Blinding was not relevant to this study. The CRISPECTOR software and expert validation relies on the knowledge of which sample is the mock and which is the treatment. The assumption is that there is no editing in the mock sample but there are sequencing errors, and in the treatment samples, there are alignment errors. Through the entire manual validation, two independent scientists examined the sequences and compared the indels to those located in the mock sample. This process is described in supplemental note S2.

Reporting for specific materials, systems and methods

We require information from authors about some types of materials, experimental systems and methods used in many studies. Here, indicate whether each material, system or method listed is relevant to your study. If you are not sure if a list item applies to your research, read the appropriate section before selecting a response.

Materials & experimental systems

n/a	Involved in the study	
☒	☐	Antibodies
☐	☒	Eukaryotic cell lines
☒	☐	Palaeontology and archaeology
☒	☐	Animals and other organisms
☒	☐	Human research participants
☒	☐	Clinical data
☒	☐	Dual use research of concern

Methods

n/a	Involved in the study	
☒	☐	ChIP-seq
☒	☐	Flow cytometry
☒	☐	MRI-based neuroimaging

Eukaryotic cell lines

Policy information about cell lines

Cell line source(s)	HEK293 and HEK293-Cas9 cell were obtained from IDT. Mobilized peripheral blood human CD34+ HSPCs purchased from AllCells, Alameda, CA.
Authentication	HEK293-Cas9 cells were confirmed by WB.
Mycoplasma contamination	Cells were not tested for mycoplasma contamination.
Commonly misidentified lines (See ICLAC register)	No Commonly misidentified lines were used in this study.
